# A Standard CMOS Humidity Sensor without Post-Processing

**DOI:** 10.3390/s110606197

**Published:** 2011-06-08

**Authors:** Oleg Nizhnik, Kohei Higuchi, Kazusuke Maenaka

**Affiliations:** Maenaka Human-sensing Fusion Project, 8111, Shosha 2167, Himeji-shi, 671-2280 Hyogo-ken, Japan; E-Mails: higuchi@eratokm.jp (K.H.); maenaka@eng.u-hyogo.ac.jp (K.M.)

**Keywords:** capacitive sensors, microsensors, humidity measurement, CMOS integrated circuits

## Abstract

A 2 μW power dissipation, voltage-output, humidity sensor accurate to 5% relative humidity was developed using the LFoundry 0.15 μm CMOS technology without post-processing. The sensor consists of a woven lateral array of electrodes implemented in CMOS top metal, a Intervia Photodielectric 8023–10 humidity-sensitive layer, and a CMOS capacitance to voltage converter.

## 1. Introduction

The history of humidity sensor integration with CMOS is at least 23 years old [1]. After nearly a quarter of a century of development, three main sensing designs have emerged in CMOS. One solution is a water-absorbing film-based design as seen in [1]. Another type of design exploits the difference in thermal conductivity between air and water vapor, as proposed in [2]. The third main design measures the dew point with a hybrid CMOS/MEMS chip, and can be used in very precise systems [3]. For mass-produced humidity sensors, like those found in air-conditioning units [4], low power dissipation and low price is obligatory. To reduce price, the amount of CMOS post-processing and packaging should be kept to minimum. However, the existing proposals for CMOS-based sensors require a porous metal on the top layer [1], or complex multilayer geometries over the wafer [5,6]. In [7], by virtue of using CMOS with the thick top metal option, only one additional layer (sensing polymer) is deposited, but then a precision mask is still needed to remove the sacrificial layer.

In the current work, we investigated the possibility of employing the standard passivation layer of the CMOS chip as the humidity sensing element with electrodes shaped similar to those described in [5,6], but using the CMOS thick top metal option in a similar fashion as described in [7]. The implementation has resulted in a functional humidity sensor with voltage output.

## 2. Circuit and Layout

**Figure 1.**
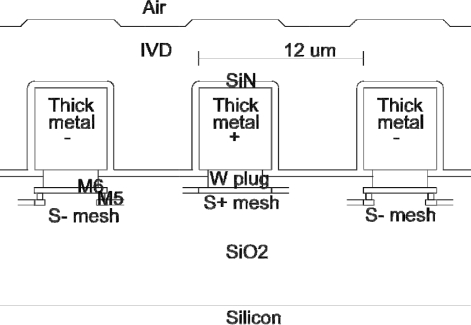
Side-view cross-section of the humidity sensor.

Sensing of humidity with hygroscopic polymer films is the most suitable approach for integrating humidity sensors in CMOS, but this approach has problems to solve. First, most hygroscopic films produce humidity-dependent capacitance, so a capacitance-to-voltage on-chip converter is necessary for simplified processing of the sensor output. Second, implementation of the sensing element is not straightforward. To obtain a large relative capacitance change, the electrical flux must be confined to the hygroscopic film. The simplest way to achieve this objective is if electrodes enclose the hygroscopic film from all sides, but for the moist air to gain access to the sensing film, a large area of the polymer must be exposed to the air. In the implemented humidity sensor, the electrical flux reaching the CMOS substrate was confined by the woven electrode mesh below the main sensing electrodes (see Figure 1). The woven mesh creates a two-dimensional multipole field distribution, with an exponentially falling intensity below the mesh. With the implemented mesh geometry, a 60% electrodes-to-substrate capacitance reduction is expected. This is more effective compared to the classical comb structure, producing one-dimensional multipole field, which is expected to close only 46% of the electrical flux lines above the substrate.

**Figure 2.**
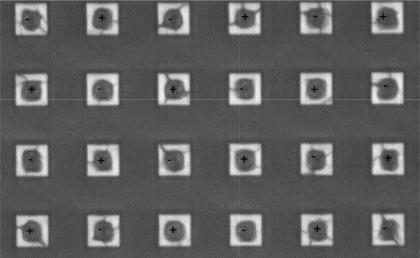
Microphotograph of the humidity-sensing surface.

Furthermore, previously proposed comb-type electrodes have a 50%metal density if sensitivity is maximized [6]. For the LFoundry 0.15 μm CMOS, the specified maximal density for the thick metal is 10%. Therefore, in the proposed design the CMOS thick metal has a density of 20%, providing a sensitivity equal to that of the classical comb pattern (see Figure 2). Positive and negative cube-like electrodes were placed in a checker-board pattern. The humidity sensing polymer was an 11 μm-thick coating of Intervia Photodielectric 8023–10, which was included in the LFoundry CMOS process as part of the pads mask. This material has a specified moisture absorption of 0.5% and a relative dielectric permittivity equal to 3.2. A sensor with comb-shaped electrodes was also manufactured, but although visually free of defects, it was found to be nonfunctional because of the short-circuits developed between electrodes.

**Figure 3.**
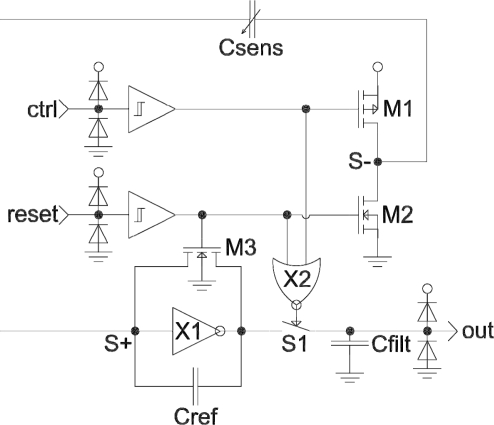
Schematic of the CMOS humidity sensor.

**Figure 4.**
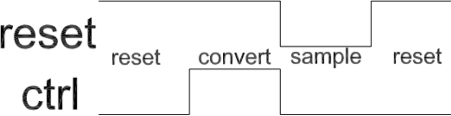
Typical input waveforms for the developed C/V converter.

A C/V (capacitance-to-voltage) converter derived from [8] was developed (see Figure 3 for schematic and Figure 4 for control waveforms). Compared to the design in [8], the proposed circuit was adapted for differential operation and a sample-and-hold circuit at output was implemented instead of the multiplexer.

## 3. Experimental Results

Experimental results from the manufactured humidity sensors are briefly summarized in Table 1.

**Figure 5.**
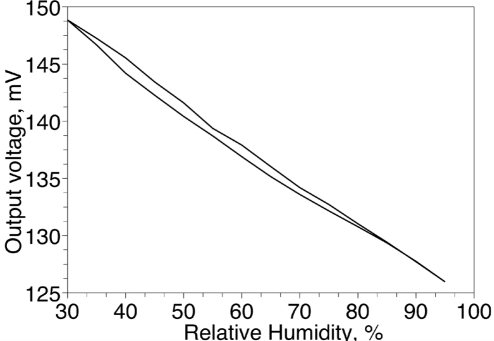
Hysteresis of humidity sensor at 25 °C.

**Figure 6.**
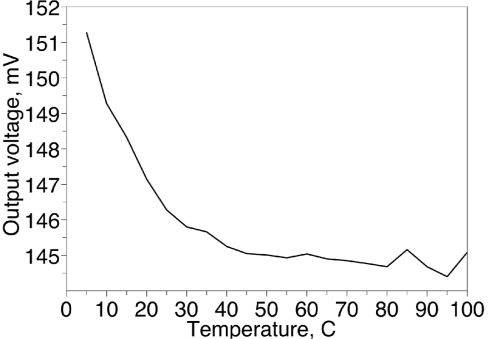
Temperature dependence of the sensor output.

Figure 5 shows typical absorption-desorption characteristic of a single humidity sensor. The hysteresis mentioned in Table 1 was found to be worst in the 45–65 °C temperature range. Figure 6 shows how output voltage of the sensor varies with the temperature. The larger temperature coefficient at temperatures below 25 °C requires some sort of the temperature compensation.

Humidity range was limited by dryer capability of the humidity chamber and surface condensation. Performance measurements were done only in the 5–100 °C range. At 100–120 °C, only reliability testing was done. The accuracy mentioned in Table 2 is defined as nonlinearity plus half the hysteresis. The 70 s response time of the designed sensor is slow compared to other designs, but still more than enough for environmental monitoring. All measurements were done using a SH-24 temperature-humidity chamber. After 52 cycles of the humidity and temperature cycling, no detectable drift was found.

## Figures and Tables

**Table 1.** Performance Summary 1st Batch of five Humidity Sensors.

**Parameter**	**Min.**	**Typ.**	**Max.**
Humidity range, RH%	10	−	95
Operating temperature range, °C	5	−	120
Hysteresis, RH%	4.4	5.5	6.6
Voltage Temperature Coefficient, RH%/°C	−0.61	0.07	0.19
Accuracy (best fit straight line), RH%	1.1	1.3	1.7
Response time, 1/e at 95% to 45% RH change, s	−	70	−
Voltage at 0% RH (extrapolated), mV	146	160	168
Gain, mV/RH%	0.34	0.37	0.39
Output impedance, MOhm	−	1	−
Power dissipation at 1V power supply, uW	1.3	1.6	1.9
Power supply voltage range, Vρ	0.8	1	1.8
Chip area with pads, mm2 (6 0.1 × 0.1 mm pads)		0.52	

**Table 2.** Comparison of the Humidity Sensors.

**Device Name**	**Power**	**Accuracy**	**Price, USD**
This work	1.6	5	1.7 (estimated)
[4] (SHT11)	28	5	36
HIH5030	540	3	6
CHS-UGS	3,000	4.5	17
